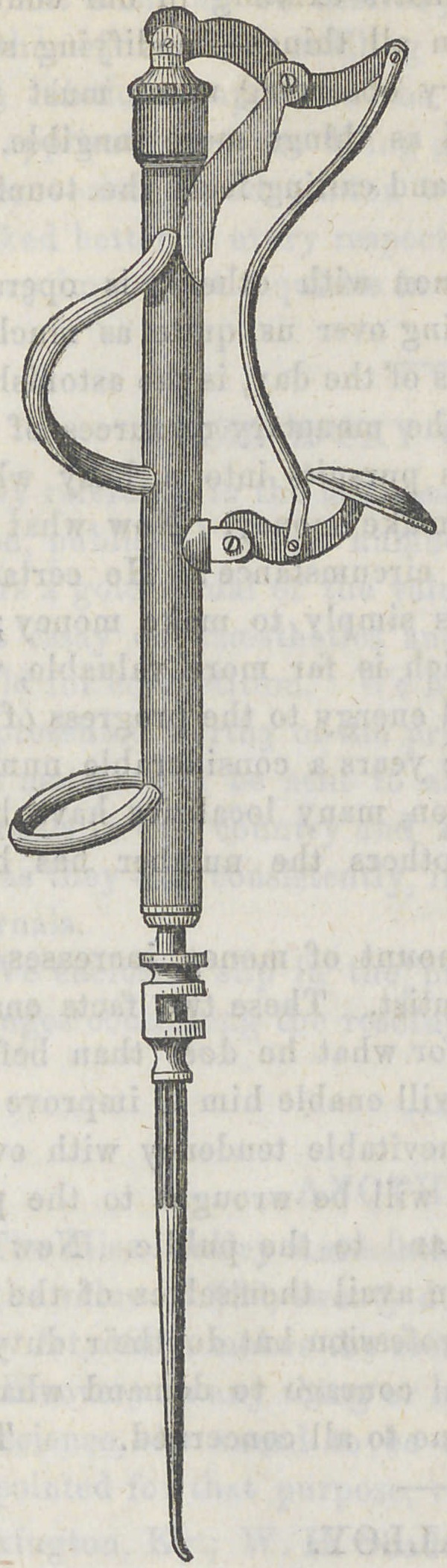# Editorial

**Published:** 1864-04

**Authors:** 


### Editorial.

### AUTOMATIC PLUGGER.

The accompanying cut represents the auatomic plugger invented by Dr. Collins:

It consists of the main cylindrical shaft, which contains two spiral springs, the hammer and the socket for the reception of the plugging point.

The socket has about 1-8th of an inch play in the cylinder, and rotates freely. The levers with their attachment for working the hammer, are clearly apparent on the engraving.

In using the instrument, the index finger of the right hand, passes through the bow at the upper part of the cylinder, and the end of the middle finger passes into the ring at the lower end, the thumb resting upon the thumb-piece, and operating the instrument by depressing this, and letting it suddenly fly back. By this means the impetus is given to the plugging point.

The instrument is very simple in structure, and doubtles, with a little practice, would be efficient in the hands of almost any one, at least we regart it as considerably in advance of any former efforts in this direction, though doubtless some improvements will be made upon this; if the instrument proves really valuable, it will, we presume, be manufactured and for sale ere long. T.

### INFLUENCE OF THE TIMES UPON THE DENTAL PROFESSION.

That the present condition of affairs existing in our country is exercising a great influence upon all things—modifying some and revolutionizing others — every observing mind must perceive, views and opinions, as well as things more tangible, are being changed ; every occupation and calling feels the touch of this all-pervading influence.

The dental profession, in common with others, is operated upon by the vicissitudes, now passing over us, quite as much as any other. One thing in the events of the day, is the astonishing and unparalleled development of the monetary resources of our country. This starts all business pursuits into a busy whirl, and men take advantage of it to make money. Now what use should the dentist make of this circumstance? He certainly should not devote all his energies simply to make money; he has the opportunity to do that which is far more valuable, viz., to devote a portion of his time and energy to the progress of his profession. Within the past three years a considerable number have withdrawn from the profession, many localities have been left without a dentist, and in others the number has been reduced.

This, together with the large amount of money, increases the demand for the services of the dentist. These two facts enable him to be far better remunerated for what he does than before, if he will but have it so, and this will enable him to improve his operations; indeed that is the inevitable tendency with every right-minded man, and thus good will be wrought to the profession, to the operator himself and to the public. Now the only question is, will the profession avail themselves of the opportunities presented? Let the profession but do their duty to their patients and have the moral courage to demand what is their due, and advantage will accrue to all concerned. T.

### WOOD'S ALLOY.

In using this material for filling, a short time since, we were forcibly impressed with the importance of absolute dryness of the cavity, by the following circumstances:

Filling a tooth in which the dentine was quite sensitive, the cavity was dried in the usual manner with flax and bibulous paper; considerable pain was experienced, and the adaptation was not such as we desired; so the filling was removed for another effort. For refilling the cavity was dried thoroughly, with bibulous paper and the warm air blow-pipe; after which the application of the filling gave but little pain—far less than before—and the adaptation was more perfect, and the material worked better in every respect; so we consider absolute dryness a very important requisite in the use of Wood's Alloy. T.

### PRIZE ESSAY ON ANÆSTHETICS.

By reference to the minutes of the Mississippi Valley Association, published in this number, it will be seen, that this body offers a gold medal of the value of one hundred dollars, for the best essay on anæsthetics and their use. It is open to the world for competition. We hope and believe that an essay will be presented worthy of the prize. The action of the Society on this matter will be sent to all the leading medical and dental journals in this country and Europe, and we hope they will, so far as they can consistently, insert the same in their respective journals.

We enclose a slip in the present number to each of our exchanges containing the resolutions in regard to the matter.

T.

### ANOTHER MEDAL.

The Miss. Valley Association proposes to give a silver medal to the value of $20, twenty dollars, to that person who, prior to Feb. 1st, 1865, makes the most valuable invention, improvement or discovery in any thing or matter pertaining to dental practice, or science, the award to be made by the following committee, appointed for that purpose, viz., Drs. A. S. Talbert, S. Driggs, Lexington, Ky.; W. H. Shadoan, Cincinnati.

We hope that all persons interested will make an effort to bring to the notice of this committee everything that would deserve their consideration. T.

### NEW DENTAL SOCIETIES.

The work of associated effort goes on. Our brethren of Chicago have organized a society with a large number of active and strong members, and they are determined to make it a permanent institution. They meet, we believe, every two weeks. tion. We have received a copy of their Constitution and By-Laws, They will doubtless have delegates in the next national association. We have received a copy of the Constitution and By-Laws in a neat form.

The profession in Louisville, Ky. have also been stirred up to a sense of their duty. we are informed they have a city society in active operation, and though we have not received anything of their proceedings we understand they are vigorously at work, and that harmony and good feeling pervades all their deliberations. T.

---

The committee appointed to solicit contributions in money, appliances, fixtures, books, &c., &c., for the Laboratory, Infirmary, Museum and Library of the College of Dental Surgery, consist of the following named persons:

COMMITTEE:

Dr. A. S. Talbert, Lexington, Ky.; Dr. S. S. White, Philadelphia, Pa.; Dr. A. M. Leslie, St. Louis, Mo.; Dr. John Allen, N. York; Dr. W. W. Allport, Chicago, Ill.; Dr. J. P. Ulrey, Rising Sun, Ind.; Dr. G. W. Keely, Oxford, Ohio; Dr. W. P. Horton, Cleveland, Ohio; Dr. Cheesebrough, Toledo, Ohio; Dr. J. W. Baxter, Warsaw, Ky.; Dr. M. De Camp, Mansfield, Ohio; Dr. G. B. Miner, Wilwaukee, Wis.; Dr. James Knapp, New Orleans, La.; Dr. I. M. Lewis, Marion, Ill.; Dr. Eli Collins, Richmond, Ind.; Dr. Joseph Richardson, Terre Haute, Ind.; Dr. David Dougherty, Danville, Ky.; Dr. M. N. Manlove, Logansport, Ind.; Dr. Geo. Watt, Xenia, Ohio; Dr. H. J. McKellops, Paris, France; the Faculty of the Ohio Dental College.

It is hoped all the stockholders will take a special interest in this matter. We see the name of S. S. White, Philadelphia, Pa., was omitted as one of the subscribers of stock. Dr. White subscribed for two shares and sent his draft for full amount.

## Figures and Tables

**Figure f1:**